# Calibration of the Epilepsy Questionnaire for Use in a Low-Resource Setting

**DOI:** 10.1155/2020/5193189

**Published:** 2020-08-31

**Authors:** Joseph O. Yaria, Adesola Ogunniyi

**Affiliations:** Department of Neurology, University College Hospital, Ibadan, Nigeria

## Abstract

**Background:**

Burden of epilepsy in sub-Saharan Africa is huge in the midst of shortage of human resource in its health sector. Using skilled staff to supervise and support lower level healthcare workers providing frontline primary healthcare is a pragmatic coping solution. But, lower level health providers face enormous challenges due to absent clinical algorithms or pragmatic rapid diagnostic tests.

**Objective:**

This study aimed to determine if the use of an epilepsy questionnaire in a traditional clinical setting would improve semiological details obtained and diagnostic accuracy.

**Methods:**

A prospective study was conducted involving patients diagnosed with epilepsy each with an eye witness who had regularly witnessed the seizures. Routine seizure history from clinical documentation and an interviewer-based questionnaire were compared. The data obtained were assessed for content, accuracy, intermethod and test-retest reliability.

**Results:**

Sixty-seven patients with a median age of 24 years were recruited. Routine seizure history had obtained less semiological details with inadequate description of nonmotor manifestations and lateralizing motor details. The questionnaire-obtained history showed higher accuracy for generalized onset seizure (0.83 vs. 0.56) and focal onset seizures (0.79 vs. 0.59). The questionnaire-obtained history also had good test-retest reliability for various semiological domains except automatisms.

**Conclusions:**

Routine seizure histories are not standardized. The use of a questionnaire goes a long way in improving semiology description in a low-resource setting and guides the health provider on what details to focus on. The use of epilepsy questionnaires should, therefore, be considered to improve semiology, especially in nonspecialist settings.

## 1. Introduction

Recent estimates suggest that about 80% of people with epilepsy worldwide live in a resource-poor setting and as many as 90% receive no form of treatment [1]. The burden of epilepsy in Africa outweighs other regions in the world [2] with over 10 million people in Africa [3] and about 4.4 million people in sub-Saharan Africa [4] suffering from epilepsy. Yet, the region suffers from marked shortage of human resource with the lowest health worker density worldwide [5, 6] and about 0.3 neurologists per million people [7, 8]. This has initiated the idea of involving skilled staff to supervise and support lower level healthcare workers who provide frontline primary healthcare [5].

In epilepsy management, the choice of medications and other interventions is dependent on the seizure type, epilepsy syndrome, and comorbidity amongst other things [9, 10]. Consequently, emphasis should be placed on seizure semiology, the circumstances under which the seizure occurred, the convulsive phase and the post-ictal state, information needed for diagnostic classification, treatment modalities, and prognosis [11–13]. This has informed innovations such as use of scoring systems [14, 15], smartphone app [16], and various contact sensors [17] to improve epilepsy diagnosis. However, these options may not be practicable for low-resource settings.

Earlier, authors have looked into the use of simple epilepsy questionnaires, especially in the low-resource setting. These questionnaires were either designed as a screening tool [18], to target the pediatric population [19], as a research tool [18], or to focus on a particular region [20]. However, most tools focused on diagnoses and not semiology details or classification. This is important in the low-income setting as studies have shown that systematically acquiring clinical information significantly increases the questionnaire performance [21, 22], especially in the absence of electroencephalography, which improved the diagnostic performance [21].

While healthcare providers in sub-Saharan Africa grapple with increased burden of epilepsy, those in primary and, probably, secondary healthcare settings face an enormous challenge in the face of absent clinical algorithms or pragmatic rapid diagnostic tests. To this end, there may be a need for establishing a widespread use of epilepsy questionnaires in the primary and secondary healthcare setting to obtain key semiology details for patient management. This is based on the premise that increased patient to doctor ratio reduces time for detailed interview, and eye-witness descriptions may also not be accurate [23–25], as key signs during seizure, especially at its onset and resolution, can help to identify epileptogenic focus in the brain [26–28] and possibly diagnosis [29]. This study aimed to determine if the use of an epilepsy questionnaire in a traditional sub-Saharan African clinical setting would improve acquisition of semiology details and diagnostic accuracy compared to routine seizure history.

## 2. Methodology

### 2.1. Study Design

A prospective study was carried out at the neurology out-patient involving patients diagnosed with epilepsy each with an eye witness who had regularly witnessed the seizures. Only patients aged over 16 years and informants who gave consent were included in the study. Participants were excluded if more than one seizure type was witnessed, they were unconscious at the time of recruitment, seizures occurred only at night, they resided alone, suffered from severe neurological impairment, e.g., mental retardation and hearing impairment, or seizures were well-controlled.

### 2.2. Variables and Their Measurements

An independent physician twice administered a predesigned questionnaire, adapted from the version designed and validated by Reutens et al. [21]—a minimum of 2 weeks apart. The questionnaire had been reported to show substantial agreement with the physician. Sensitivity and specificity for generalized seizures were as high as 1.00 and 0.93, respectively, and 0.88 and 1.00, respectively, for partial seizures [21]. This tool was selected because it was validated for adults, contained relevant semiology details, allowed for verbatim records of seizure description by both the patient and informant, and had a better psychometric scores than the recent Bayesian tool by Patterson et al. [30]. However, both tools had similar questions. Data obtained from the administered questionnaire was interpreted by JY and confirmed by an independent neurologist.

Details of seizure semiology were also extracted from medical records, and this information made up the data analyzed for routine seizure history. Eye witness was also required to identify motor manifestations that best describe motor semiology from a video compilation. Almost all patients recruited had an EEG performed primarily to assist seizure diagnosis, classification, and possibly localizing the epileptogenic zone. Two EEG trained physicians and one technologist interpreted the EEG result. Participants returned for a repeat interview 2–4 weeks later, after which they completed the study.

For this study, the criterion for diagnosis and classification was based on a predefined algorithm which combined clinical history [31], EEG results, video evidence, and neuroimages. At least, two trained neurologists reviewed this information separately and determined seizure classification according to the 2017 ILAE criteria [32]. See the supplementary file (Available here). The screening tool designed by Ali et al. was used to determine nonepileptic seizures [33]. Based on the 2013 ILAE Nonepileptic Seizure Task Force recommendation, the diagnostic level of certainty for PNES in this study was at best possible or probable [34].

### 2.3. Data Analysis

STATA statistical software package (Stata® release 12, 2011) was used for data analysis after variables were examined for missing data and outliers prior to analysis of the data. Semiology details were based on the ILAE 2017 criteria [32]. Impaired awareness was defined by the presence of any of the following: amnesia of event, blank spell, or loss of consciousness. Motor sphere was made up of myoclonus, tonic posturing, clonus, version, hyperkinetic movement, and automatic activity. Versive activity was defined as an unquestionably forced and involuntary sustained unnatural positioning of the head and eyes [35].

Fisher's exact test was used to compare the proportion of the obtained semiology features between the routine seizure history and questionnaire-obtained history. Fisher's exact test instead of McNemar (despite the paired data) was used, as the objective was not consistency of response with various raters but difference in the amount of obtained response. For seizure classification, sensitivity, specificity, and diagnostic accuracy were calculated and reported. Intermethod and test-retest reliability were assessed by calculation concordant conclusion, Cohen's kappa, and applying the exact McNemar's test to assess consistency [36].

## 3. Results

### 3.1. Baseline Description

Sixty-seven patients with a median age of 24 years (range: 16–76 years) and their informants, median age of 47 years (range: 18–76 years), were recruited. The patients comprised 28 (45.9%) females and 33 (54.1%) male patients. The median age of seizure onset was 18 (range: 1–76) years, and median age of epilepsy diagnosis was 21 (range: 2–76) years. Thirteen (21.7%) participants had a positive family history of epilepsy while 46 (76.7%) were on AEDs, with carbamazepine being the most prevalent 28/46 (62.2%). Of the participants, 10 (16.7%) admitted to learning difficulty.

During routine seizure history, only 28 (45.2%) patients were questioned about nonmotor symptoms, 2 (3.2%) about autonomic symptoms, and 33 (53.3%) concerning impaired awareness (these included blank spells, amnesia of event, and unawareness during event), and motor details were obtained in 55 (88.8%) and post-ictal features in 36 (58.1%) patients. Dramatic motor manifestations as clonus, 48 (77.5%) and tonic manifestations, 36 (61.3%) were the highest motor details obtained from routine history.

### 3.2. Distribution of Seizure Semiology

#### 3.2.1. Nonmotor Focal Manifestation

**Figure 1:**
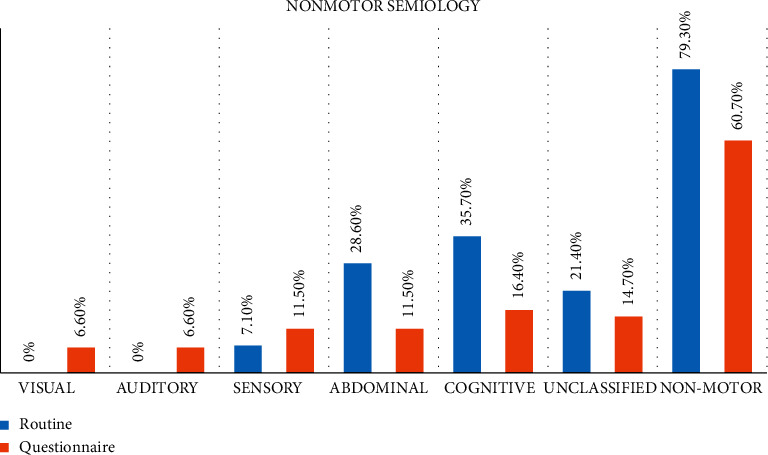
Proportion of participants with various nonmotor focal onset semiology obtained from the routine seizure history (blue) and questionnaire administration (red). The denominators used to calculate proportion were based on numbers of respondents who were interviewed. However, with the routine seizure history, not all domains were frequently explored.

Based on seizure description obtained via routine clerking, there was a higher prevalence of nonmotor focal onset, 23 (79.3%). The most common nonmotor presentation was cognitive, 10 (35.7%). In contrast, the questionnaire-obtained history showed 37 (60.7%) had nonmotor focal onset with 10 (16.4%) having cognitive manifestation (see Figure 1).

#### 3.2.2. Awareness

Based on routine seizure history, 28 (87.5%) had symptoms in keeping with impaired awareness. However, the questionnaire-obtained history reported a higher count with 53 (86.9%) having symptoms in keeping with impaired awareness (see Table 1).

#### 3.2.3. Motor Sphere

**Figure 2:**
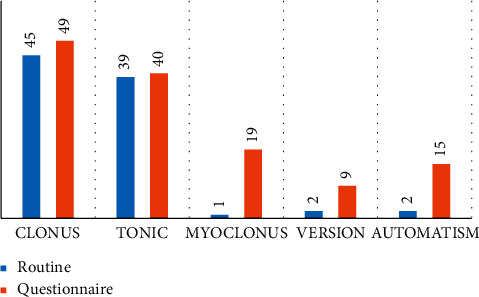
Number of participants with various motor semiology from the routine seizure history (blue) and questionnaire administration (red). The numbers were used to plot the graph due to missing values, especially with routine seizure history, where not all domains were frequently explored. There was 1 (1.6%) case of atonic activity and complex automatism described as a cycling motion.

Comparing the routine seizure history to questionnaire-obtained history, there were similar rates for clonic activity. However, just one participant had myoclonus activity, and two had automatism and versive movements documented from the routine seizure history as opposed to 19 (31.7%), 15 (24.6%), and 9 (14.7%) from questionnaire obtained seizures, respectively (see Figure 2). Comparing the routine history and questionnaire history to video selections, the questionnaire history had better percent agreement and kappa estimates as shown in Table 2.

### 3.3. Seizure Classification

Based on the available information from the routine history, a higher proportion was classified as generalized onset seizures, 29 (47.5%) as opposed to 13 (21.3%) from the questionnaire-obtained history (see Table 3). There was suboptimal agreement between the routine seizure history and questionnaire-obtained history with 52.4% agreement, kappa: 0.10, and McNemar *p*: 0.012. The routine seizure history had worse classification accuracy when compared to the questionnaire-obtained history (see Table 4).

### 3.4. Test-Retest Reliability

Of the recruited patients, 48 (78.7%) returned for second assessment. However, those who did not return for a repeat assessment and those who did had similar sociodemographic and epilepsy characteristics. The percent agreement was almost perfect (>80%) except for blank spell (74.5%). Kappa coefficients were varied ranging from 0.33–0.93. Awareness had the highest kappa coefficient of 0.93, automatism having the lowest kappa coefficient, 0.33, followed by blank spell, 0.42, as shown in Table 5.

## 4. Discussion

The quest for diagnostic certainty in adult medicine is largely a function of the availability and characteristics of treatment options and prognosis. The poor specificity of AEDs implies that substantial diagnostic uncertainty can be tolerated. However, in adult medicine, seizure diagnosis and classification guides the physicians to the next step in management. Findings from this study point out potential pitfalls of the routine seizure history in our environment. Despite fair reliability, its content and accuracy are suboptimal. Result from this study showed the routine seizure history to focus more on the motor manifestation,mainly the tonic and clonic activity, compared to other semiology details. As a result, using strict criteria up to 63.9% of the participants recruited could not be properly classified or seizure focus localized due to the absence of adequate semiology description, mainly regarding nonmotor focal seizures. This is similar to the findings of Bodensteiner et al. who reviewed 2219 seizure documents and reported poor descriptions by physicians resulting with about 22–51% of those seizures unclassifiable [37]. Wulf, in a study evaluating seizure observation and documentation, also noted that more details are documented for motor description [38].

This flaw does not rest only with the physician as Dash et al. also noted that semiological features of motor activities had the maximum yield from the witness [39]. It is expected that the motor manifestation of a seizure is what the informant focuses on as it is the most obvious, dramatic, and worrying feature of a seizure. However, responsibility of teasing out other semiology details lies with the physician. This is of added importance in SSA and other low-resource settings where the clinical history might be the only basis for diagnosis.

According to Stelly and Goldstein, missing information may indicate that patients do not understand questions asked, irrelevant questions are asked, or the physicians are not asking necessary questions [40]. However, when less than half of the medical records reviewed, as seen in this study, had documentation on nonmotor focal seizures, autonomic, and conscious state, one wonders if these spheres were explored by the physician. The fact that the informants provided this information when the questionnaire was administered further strengthens this notion. This was a view held by Reutens et al. which informed their advocacy of the use of a questionnaire to solve the problem of incomplete history taking [21].

It is also worth noting that while motor sphere was the most characterized, motor details that could assist in lateralization, localization, and ultimately management implications were not routinely asked for. Dystonic posturing, automatisms, version, myoclonus, and other post-ictal details were rarely documented with more focus on determining if the tonic and clonic manifestation were generalized or not. This was also reported by Mannan and Wieshmann [24] and Heo et al. also [25] where lateralizing features, unresponsiveness, automatism, and motionless staring are frequently missed. It is, therefore, not surprising that a few local studies reported a higher prevalence for generalized onset epilepsy [41]. However, a possibility that these information was obtained by the consulting physician in making his clinical decision, however, not being documented in the medical records should be considered.

The suboptimal accuracy of the routine seizure history has been reported by various authors [23, 42, 43] though none compared the accuracy of routine description to the questionnaire. Heo et al. [25], Dash et al. [39], and Seneviratne et al. [44] reported falsely elevated prevalence for generalized seizure using the routine clinical history. A finding that was not surprising in this study group as nonmotor focal seizures is poorly characterized in the routine seizure history. The difference in seizure classification for 47.6% participants between the routine and questionnaire-based history is notable and worrisome. Deacon et al. reported dissimilar results with an accuracy estimate of 0.94 for routine clinical descriptions [45]. But, the sample population used was highly selective as only patients with refractory temporal lobe seizures were recruited, thereby limiting generalization of the result. Hirfanoglu et al. [46], Rugg-Gunn et al. [23], and Jin et al. [47] reported contradictory findings, but obvious methodological variations can explain the disparity. Hirfanoglu carried out their study only on children, while Rugg-Gunn et al. designed their study in a controlled laboratory environment.

A notable finding in this study was the wide differences between percentage concordance and kappa estimates which suggested an element of chance. Since the responses are dichotomous in nature, a high reliability is likely. However, guessing is also likely to occur, which led to Cohen's creation of the kappa value to control for chance [48]. Once again, inadequate information as to seizure description may be a plausible explanation for poor reliability as there is a chance for conjecture. Findings from Bodensteiner et al. support this argument. They reported low overall agreement between observer pairs (kappa: 0.24–0.38) after using various neurologists to classify seizure based on clinical documentation. However, on performing a restricted analysis including only medical records with a fair degree of detail, there was an improvement in the kappa value. This adds credence to the hypothesis that these documentations lacked adequate information for classification [37].

However, laying the blame solely on information adequacy may be too simplistic. A look into other areas to help unravel the discordance in seizure classification is necessary. Based on the result of McNemar testing, the errors noted with individual semiology description were random and not systematic, thus suggesting that the art of routine seizure history itself is not standardized. Physicians' interpretation of obtained descriptions can be subjective. A phenomenon may explain poor reliability of nonmotor focal seizures phase, the most subjective sphere of seizure semiology. Even the informants's impression of the examining physician and the environment in which the history is being obtained amidst other factors come to play in accuracy and reliability of the routine seizure history. This ultimately leads to consistently faulty seizure classification which explains the significant McNemar test with comparison of seizure classification. The use of a questionnaire may help reduce physician-related factors, e.g., variation in interpretation of informants' description or variety in the use of words when framing questions [49]. It should be noted that there was better intermethod reliability when the questionnaires-based history was compared to video recordings than when the routine history was compared to video recording buttressing the point.

The key to obtaining a more accurate classification from the routine seizure history may actually lie in improving its content quantity and quality. This has led to researchers exploring various methods with a view to improving epilepsy accuracy. The use of questionnaires [21, 30], video devices [39, 46, 50], repeated viewing of an event [47], and specialty diagnosis [47] are various options explored. It should also be said that the inability to correlate the seizure history with intracerebral activity in this study adds a limitation to the conclusion that can be made.

### 4.1. Study Limitation

A few limitations were encountered in the study with the lack of a better reference standard or criteria for diagnosis and classification being the notable one. The preferred options include VEEG, ictal EEG, or functional imaging, none of which are readily available in our environment. It is, however, felt that the algorithm used would closely appropriate the true picture as multiple investigative modalities and repeated seizure accounts were combined to come to a conclusion. Also, the routine seizure history was largely based on medical documentation. There is a possibility that not all of the seizure details obtained from informants were documented in their medical records. Other options such as an audio recording of routine seizure might have been a more appropriate method, but it was felt that it would introduce a bias as the clinician would be conscious of being recorded. Lastly, the use of kappa estimate to account for chance has its limitations as the response with the routine seizure history was skewed. Calculation of other alternatives such as the Bryt et al. and phi statistic have been recommended as they are resistant to skewed responses. But, they are uncommonly reported in medical statistics and clinical interpretation may be queried; hence, they are not used for this study.

## Figures and Tables

**Table 1:** Participants with impaired awareness during seizure. The denominators used to calculate the proportions was not identical due to missing values especially with routine seizure history were not all domains were frequently explored.

	Routine seizure history	Questionnaire-obtained history
Amnesia	9 (100.0)	53 (86.9)
Blank spell	4 (66.7)	18 (29.5)
Obvious of consciousness	24 (85.7)	50 (82.0)
Impaired Awareness	28 (87.5)	53 (86.9)

**Table 2:** Result of intermethod reliability comparing variables obtained by the routine seizure history and questionnaire-obtained history to the video selection. The McNemar test was performed to assess consistency with the *p* value reported.

	Routine		Questionnaire	
	% agreement	Kappa	% agreement	Kappa
Consciousness				
Blank spell			82.0	0.53
Motor				
Clonus	85.7	0.39	^2^85.3	^2^0.62
Tonic	78.7	0.37	90.2	0.78
Myoclonus			81.7	0.58
Version	66.7	0.04	90.2	0.68
Automatisms			95.0	0.86
Seizure classification	^4^56.5	^4^0.40	81.3	0.46

^2^McNemar *p* value: 0.020. ^4^McNemar *p* value <0.001.

**Table 3:** Seizure classification from various methods.

	Routine	Questionnaire	Standard
Focal onset, *N* (%)	5 (8.2)	5 (8.2)	5 (8.2)
Focal to bilateral	16 (26.2)	30 (49.2)	39 (63.9)
Generalized, *N* (%)	29 (47.5)	13 (21.3)	10 (16.4)
^*∗*^PNES, *N* (%)	—	—	3 (4.9)
Unclassified, *N* (%)	11 (18.0)	13 (21.3)	4 (6.6)

^*∗*^PNES: psychogenic nonepileptic seizure.

**Table 4:** Summary of the accuracy of the routine and questionnaire-obtained history.

	Accuracy	Sensitivity	Specificity
Focal onset			
^1^Routine	0.59	0.45	0.94
^2^Questionnaire	0.79	0.75	0.88

Generalized onset			
^3^Routine	0.56	0.60	0.55
^4^Questionnaire	0.83	0.60	0.86

^1^ *p* value: 0.002. PPV: 0.95. NPV: 0.40. ^2^*p* value <0.001. PPV: 0.94. NPV: 0.58. ^3^*p* value: 0.002. PPV: 0.21. NPV: 0.87. ^4^*p* value <0.001. PPV: 0.46. NPV: 0.92. Algorithm Used for Classification Standard in Appendix VII.

**Table 5:** Result of test-retest reliability of variables obtained by the questionnaire-obtained history. The McNemar test was performed to assess consistency with the *p* value reported.

	% Agreement	Kappa	*p* value
Cognitive sphere	83.7	0.65	0.289
Visual	91.8	0.46	1.000
Sensory	89.8	0.49	1.000
Abdominal	95.9	0.78	0.500
Psychic	85.7	0.54	1.000
Unclassified	89.8	0.61	1.000
Autonomic sphere	92.0	0.56	0.625
Conscious sphere			
Blank spell	74.5	0.42	0.581
Unaware	98.0	0.93	1.000
Amnesia	95.8	0.73	0.500
Motor			
Clonus	92.0	0.77	1.000
Tonic	91.7	0.79	0.125
Myoclonus	85.4	0.64	0.453
Version	90.2	0.66	0.625
Automatisms	80.0	0.33	0.344
Seizure classification	89.5	0.75	0.625

## Data Availability

The authors confirm that the summary data supporting the findings of this study are available with its supplementary file with raw data available from the corresponding author (J.O.Y) on request.

## Additional Points

*Paper Context*. Authors have underscored dangers of incomplete history taking in seizure management. This has led to the design of seizure questionnaires. However, in sub-Saharan Africa, most questionnaires are designed for research purposes. This study assesses if a seizure questionnaire can be used in a routine clinic in a low-resource setting. It is believed this would lead to improvement in seizure diagnosis via implementation of standardized questionnaires as part of integrated epilepsy care involving the primary healthcare level.

## Supplementary Materials

Supplementary Materials The authors confirm that the summary data supporting the findings of this study are available within its supplementary file with raw data available from the corresponding author (J.O.Y) on request.
