# Supplementary material for: Calibration of the Epilepsy Questionnaire for Use in a Low-Resource Setting
Source: J Environ Public Health. 2020 Aug 31;2020:5193189. doi: 10.1155/2020/5193189 (PMC7479480; doi:10.1155/2020/5193189)
Supplement: Supplementary Materials — The authors confirm that the summary data supporting the findings of this study are available within its supplementary file with raw data available from the corresponding author (J.O.Y) on request. [file 5193189.f1.docx]

**CALIBRATION OF EPILEPSY QUESTIONNAIRE FOR USE IN A LOW-RESOURCE SETTING**

Joseph. O. Yaria^1^ (MBBS, FMCP (Neurol), Adesola Ogunniyi^1^ (FWACP, FRCP)

^1^Department of Neurology, University College Hospital, Ibadan, Nigeria.

Prof. A. Ogunniyi: aogunniyi53@yahoo.com

Corresponding Author

Dr. Joseph Yaria

Department of Neurology

University College Hospital, Ibadan, Nigeria

E-mail: jyaria@yahoo.com

+234 802 849 2054

Running title: Use of Epilepsy Questionnaire in Low Resource Setting

Keywords: Semiology. Accuracy. Seizure. Reliability. Primary Healthcare. Secondary Healthcare

# SUPPLEMENTARY FILE

Table 1: Proportion of Participants with Documented Content in Various Spheres

|  | Routine | Questionnaire | P-Value |
| --- | --- | --- | --- |
| Aura Sphere, N (%) |  |  |  |
| Enquiry | 28 (45.2) | 61 (100.0) | <0.001 |
| *Description | 18 (94.7) | 37 (100.0) | <0.001 |
| *Duration | - | 33 (89.2) | <0.001 |
| Autonomic Sphere, N (%) |  |  |  |
| Identify | 2 (3.2) | 61 (100.0) | <0.001 |
| *Description | 2 (100.0) | 6 (100.0) | - |
| Conscious Sphere, N (%) |  |  |  |
| Blank Spell | 6 (9.8) | 61 (100.0) | <0.001 |
| Unaware | 28 (45.9) | 61 (100.0) | <0.001 |
| Amnesia | 9 (14.7) | 60 (98.4) | <0.001 |
| Motor, N (%) |  |  |  |
| Clonus | 49 (80.3) | 61 (100.0) | <0.001 |
| Tonic | 47 (77.1) | 61 (100.0) | <0.001 |
| Myoclonus | 2 (3.3) | 60 (98.4) | <0.001 |
| Version | 19 (31.2) | 54 (88.5) | <0.001 |
| Automatisms | 2 (3.3) | 61 (100.0) | <0.001 |
| Region | 48 (77.5) | 61 (100.0) | <0.001 |
| Duration | 21 (34.5) | 61 (100.0) | <0.001 |
| Hyper-motor |  |  |  |
| Post-Ictal, N (%) |  |  |  |
| Description | 34 (55.7) | 60 (98.4) | <0.001 |
| Duration | 7 (11.5) | 45 (73.8) | <0.001 |
| Presence of deficit | 5 (8.2) | 60 (98.4) | <0.001 |

*Of those who admitted to having interested variable

**Clinical Questionnaire.**

**Calibration of Seizure History.**

**Interviewer Based Questionnaire**

This is a research on accuracy of seizure history. Kindly sign at the bottom if you consent.

| Serial number |  | Hospital number |  |
| --- | --- | --- | --- |
| Age |  | Gender (circle appropriate) | 1.Male 2.Female |
| Address (City, State) |  | Marital Status | 1.Single 2.Married 3.Separated |
| Religion | 1.Christian 2.Islam 3.Others | Tribe | 1.Yoruba 2.Igbo 3.Hausa 4.Others |
| Phone number |  | Educational Status | None 1^O^ 2^O^ 3^O^ 4^O^ |
| **Witness Details.** |  |  |  |
| Relationship to Patient | 1.Parent 2.Sibling 3.First Degree Relative 4.Second Degree Relative 5.Friend | | |
| Age |  | Gender | 1.Male 2.Female |
| Marital status | 1.Single 2.Married 3.Separated | Religion | 1.Christian 2.Islam 3.Others |
| Educational Status | None 1^O^ 2^O^ 3^O^ 4^O^ |  |  |

|  | Patient’s Response |  |
| --- | --- | --- |
| 1 | Have you ever heard of epilepsy? | 0. No 1. Yes |
| 2 | Have you ever observed an epileptic fit? | 0. No 1. Yes |
| 3 | Do you have a relative who suffers from epilepsy? | 0. No 1. Yes |
| 4 | What do you think causes epilepsy? | 0. Wrong 1. Correct |
| 5 | Do you think epilepsy/seizures is a contagious disease? | 0. No 1. Yes |
| 6 | What will you do if some has a seizure now? | 0. Wrong 1. Correct |
| 7 | Do you think epileptic fits can kill? | 0. No 1. Yes |
|  | Witness Response |  |
| 8 | Have you ever heard of epilepsy? | 0. No 1. Yes |
| 9 | Have you ever observed an epileptic fit before this patient? | 0. No 1. Yes |
| 10 | Do you have a relative who suffers from epilepsy? | 0. No 1. Yes |
| 11 | What do you think causes epilepsy? | 0. Wrong 1. Correct |
| 12 | Do you think epilepsy/seizures is a contagious disease? | 0. No 1. Yes |
| 13 | What will you do if some has a seizure now? | 0. Wrong 1. Correct |
| 14 | Do you think epileptic fits can kill? | 0. No 1. Yes |
|  | SEIZURE HISTORY |  |
| 15 | Age of first seizure |  |
| 16 | Age of first diagnosis |  |
| 17 | Is there anyone in your family with similar complains | 0. No 1. Yes |
| 18 | If yes, Who |  |
| 19 | Are you on any anti-epileptics? | 0. No 1. Yes |
| 20 | If yes, what is/are the name(s) |  |
| 21 | How long have you been on medication? |  |
| 22 | Was there any developmental delay as a child? | 0. No 1. Yes |
| 23 | Have you noticed any learning disability? | 0. No 1. Yes |
|  | ELECTRO-ENCEPHALOGRAPHY |  |
| 24 | Background waves |  |
| 25 | Spike Location |  |
| 26 | Sharp Location |  |
| 27 | Diagnosis. |  |
| 28 | Rank of Physician: |  |

Use the space provided below for any additional comments.

[Comments]

**Semiology Questionnaire**

**Calibration of Seizure History**

**Interviewer Based Questionnaire**

Your opinion is important to us. Additional comments and suggestions are encouraged.

| Hospital number: |  | Department |  |
| --- | --- | --- | --- |
| Supervisor (optional) |  | Position |  |

| 1 | How many types of seizures do you think you have? |  |
| --- | --- | --- |
| 2 | Please describe what happens in each type of seizures. |  |
| 3 | Do you have blank spells or spells where you just switch off? | 0. No 1. Yes |
| 4 | Do you have spells in which you twitch or jerk, especially just after waking up? | 0. No 1. Yes |
|  | COGNITIVE SPHERE |  |
| 5 | Do you have any warning before seizures occurs? | 0. No 1. Yes |
| 6 | Describe the warning? (circle appropriate)   1. Paresthesia (select starting dermatomes and its spread) 2. Flashing colors, dark dots or blindness, visual hallucinations (select visual field) 3. Distorted objects, distorted sounds, things are in slow motion or fast forward 4. Buzzing noise, hearing sounds 5. Offensive smell, funny taste 6. Nausea, epigastric discomfort, mouthwatering or drooling 7. Flashbacks, feeling of being in a dream, fear 8. Anger, happiness, pleasure |  |
| 7 | Does the warning ever occur on its own? | 0. No 1. Yes |
| 8 | How long does these warnings last? |  |
|  | AUTONOMIC SPHERE |  |
| 9 | Do you notice any of the following: very fast pulse, pounding heart, marked sweating, change in color of the cheeks? | 0. No 1. Yes |
| 10 | Do they happen before the jerky movements? | 0. No 1. Yes |
|  | CONSCIOUSNESS SPHERE |  |
| 11 | Are you aware of what is going on around you during the seizure? | 0. No 1. Yes |
| 12 | If yes to question 9, how long are you blank? |  |
| 13 | Do you retain some awareness of your surroundings or you go completely blank? | 0. No 1. Yes |
| 14 | Do you stop what you are doing or you continue automatically (like a robot)? | 0. No 1. Yes |
|  | MOTOR SPHERE |  |
| 15 | Have your legs given way or you dropped to the ground without losing consciousness? | 0. No 1. Yes |
| 16 | Have your arms dropped or sagged without losing consciousness? | 0. No 1. Yes |
| 17 | Have you dropped objects without losing consciousness? | 0. No 1. Yes |
| 18 | Does your head drop or sag without losing consciousness? | 0. No 1. Yes |
| 19 | Which directions do the eyes look? | 0. Left 1. Right |
| 20 | How long does the eye rolling last? |  |
| 21 | Which direction does the head turn? | 0. Left 1. Right |
| 22 | How long does it last? |  |
| 23 | Are the arms held stiff in a particular position? | 0. No 1. Yes |
| 24 | Describe what happens with arms? (say what direction) |  |
| 25 | Are both arms or is just one arm affected? | 0. Both 1. One |
| 26 | How long does it last? |  |
| 27 | Are the legs held stiff in a particular position? | 0. No 1. Yes |
| 28 | Describe what happens? (say what direction) |  |
| 29 | Are both legs or is just one leg affected? | 0. Both 1. One |
| 30 | During seizure, does breathing stop? | 0. No 1. Yes |
| 31 | Is there any jerking or twitching of the face, arms or legs | 0. No 1. Yes |
| 32 | Which parts of the body do they affect? |  |
| 33 | Does the jerking affect both sides of the body at the same time or only one side at a time? | 0. Both 1. One |
| 34 | Are the jerks infrequent and irregular, or are they repeated and regular? (circle as appropriate) |  |
| 35 | How long does the jerking last? |  |
| 36 | During a seizure, do the eyelids twitch or repeated blinks? | 0. No 1. Yes |
| 37 | How long does it last? |  |
| 38 | Do any of the following occur? Smacking of lips, licking of lips, chewing, swallowing, laughing, picking at or fiddling with things, walking or making stepping or bicycling movements, rubbing finger, speaking? (circle as appropriate) |  |
| 39 | Do you ever bite your tongue? | 0. No 1. Yes |
| 40 | Do you wet yourself during a seizure? | 0. No 1. Yes |
|  | POST-ICTAL PHASE |  |
| 41 | After seizures are you confused, drowsy, and tired or sleep? (circle as appropriate) |  |
| 42 | How long does this last? |  |
| 43 | After seizure, do you have a headache? | 0. No 1. Yes |
| 44 | Is the headache on one side of the head or all over? | 0. All 1. One |
| 45 | Describe it |  |
| 46 | Do seizures have any longer lasting effects on vision, speech, sensation or muscle power? | 0. No 1. Yes |
| 47 | Describe it |  |
| 48 | How long does this effect last? |  |
| 49 | Do you remember the seizure? | 0. No 1. Yes |

**Algorithm for classification**

Focal lesion seen, Correlates with seizure description

STEP 1: Neuro- Imaging

None Seen

STEP 2: EEG

None Seen

Focal discharge

None Seen

Localization related

STEP 3: Aura Related

**FOCAL ONSET SEIZURE**

STEP 4: Dialeptic Features

None Seen

At least one present

None Seen

STEP 5: Motor Features

No Features of Focality

Features of Focality

**FOR REVIEW**

**GENERALIZED**

**FOCAL TO BILATERAL TONIC-CLONIC**

**FOCAL ONSET SEIZURE**

**Details of Participants**

|  | classification | key semiology | EEG | Neuro  imaging | CASENOTE DOCUMENTATION |
| --- | --- | --- | --- | --- | --- |
| 1 | PNES | Panting after Emotional Situations | Normal |  | Hyperventilation then LOC |
| 2 | Focal Onset | Tonic Neck Twisting |  |  | Tonic neck twisting. Jerks |
| 3 | Focal Onset | Facial Grimacing and Twitching | Polymorphic Delta | Opercular Infarct | Abnormal oral movement |
| 4 | Focal, Bilateral | Headache. Faints. | Focal, Bilateral |  | Sudden collapse |
| 5 | Focal, Bilateral | Paresthesia. Automatism. TCM | Focal. FIRDA |  | Paresthesia. Tonic. TCM. Violence |
| 6 | Focal, Bilateral | Left Clonus. Speech Arrest, Unaware | Focal |  | Headache. TCM |
| 7 | Focal, Bilateral | Dizziness, right TCM, Unaware |  | Frontal Infarct | Right TCM. |
| 8 | Focal, Bilateral | Dizziness, scream then fall | Focal |  | TCM |
| 9 | Focal, Bilateral | TCM. Dystonic Hand | Focal, Bilateral |  | Sudden Cry. TCM. |
| 10 | Focal, Bilateral | Nausea, TCM. Dystonic Hand. | Focal |  | Oral Movement. Right TCM |
| 11 | Generalized | Loud Cry. TCM | Non-specific spikes |  | TCM |
| 12 | Focal, Bilateral | Throat itch, collapse | Focal | Normal | Throat sensation. Fall |
| 13 | Focal, Bilateral | Anger, TCM. Figure 4 Posturing | Focal, Bilateral |  | Right Jacksonian TCM |
| 14 | Focal, Bilateral | TCM. Dystonic Hand | Focal |  | Yell then TCM |
| 15 | PNES | Tears during ictus. Jerks | No ED |  | Generalized Jerks |
| 16 | PNES | Prolonged malaise. Trembling | No ED |  | Left Jacksonian TCM |
| 17 | Focal, Bilateral | Fear. TCM | Focal, Bilateral | Cortical Atrophy | Fear. TCM |
| 18 | Focal, Bilateral | Automatism, TCM | Focal, Bilateral |  | Dizzy. Right Jacksonian TCM |
| 19 | Focal, Bilateral | Fear, Amnesia, TCM | No ED |  | Generalized Tonic |
| 20 | Focal, Bilateral | TCM. Dystonic Hand | Focal, Bilateral |  | Blank Spell. General Clonic |
| 21 | Focal Onset | Heat, Dizzy | No ED |  | Abdominal Upset. Chest Tightness. Dizziness |
| 22 | Focal, Bilateral | Aggression, TCM | Focal, Bilateral |  | Aggression. TCM |
| 23 | Focal Onset | Fear, Jacksonian TCM | Focal, Bilateral |  | Fear. Left Jacksonian TCM |
| 24 | Focal, Bilateral | Speech Arrest, Atonia | Focal | Frontal GBM | Abdominal. Automatism. |
| 25 | Focal, Bilateral | Automatism. Cry. TCM |  |  | Abdominal. TCM |
| 26 | Generalized | No Aura. TCM |  |  | Dizziness. Forgetful. TCM |
| 27 | Focal, Bilateral | Dream State, Blank Stare, Behavioral Outburst | Focal | Temporal Sclerosis | Behavioral Outburst |
| 28 | Focal, Bilateral | Automatism. TCM. Fencing | Focal | Post Gunshot Injury | Sudden Scream then TCM |
| 29 | Focal, Bilateral | Trance. Right Jacksonian TCM |  | Temporal Meningioma | Right UL TCM then LOC |
| 30 | Focal, Bilateral | Flashing colors, Cry. General Tonic | Focal |  | Blank Spell. TCM |
| 31 | Focal, Bilateral | TCM | Focal, Bilateral | Normal | TCM |
| 32 | Focal, Bilateral | Dyspepsia. Automatism. TCM | Generalized |  | Nausea. Amnesia. TCM |
| 33 | Generalized | Loud Cry. TCM |  |  | Loud cry then TCM |
| 34 | Focal, Bilateral | Dyspepsia, TCM | Focal, Bilateral |  | TCM |
| 35 | Generalized | TCM |  |  | Loud cry then TCM |
| 36 | Focal, Bilateral | Blank Stare. TCM |  |  | TCM |
| 37 | Focal, Bilateral | Stares. Robotic Walk. TCM |  |  | TCM |
| 38 | Generalized | Sudden arrest. Stares | Generalized. JAE |  | Stares. Myoclonic Jerk |
| 39 | Focal, Bilateral | Dyspepsia, Automatism, TCM | Focal, Bilateral |  | TCM |
| 40 | Focal, Bilateral | Warm. TCM. Dystonic Hand | Generalized. |  |  |
| 41 | Generalized | Stares | Generalized. FIRDA |  | Blank Stare |
| 42 | Generalized | Generalized Jerks | JME |  |  |
| 43 | Focal, Bilateral | Dyspepsia, TCM |  |  | Limb Jerk. LOC |
| 44 | Focal | Dreamy. Behavior. TCM | Focal, Bilateral |  | Limb Jerk. LOC |
| 45 | Focal, Bilateral | Automatism, Unaware | No ED |  |  |
| 46 | Focal, Bilateral | Vertiginous. Automatism. TCM |  |  | TCM |
| 47 | Focal, Bilateral | TCM, Unaware | Generalized |  | TCM |
| 48 | Focal, Bilateral | Visual Distortion, General Tonic | Focal, Bilateral |  |  |
| 49 | Focal, Bilateral | Dreamy, Generalized Dystonia | Focal, Bilateral |  | Aura then TCM |
| 50 | Focal, Bilateral | Fencing. One-sided Dystonia, Unaware | Focal |  | Sudden fall then left Clonus |
| 51 | Focal Onset | Paresthesia, Cycling Motion | Focal |  |  |
| 52 | Focal, Bilateral | Versive. TCM | Generalized |  | Jacksonian TCM |
| 53 | Focal, Bilateral | Sweat. Aggression. Jerk. TCM | Focal | Normal | Aggression. Palpitation |
| 54 | Focal, Bilateral | Vertiginous. Automatism. TCM | Non-specific |  | TCM |
| 55 | Generalized Onset | Generalized TCM | Right Temporal Theta |  | TCM |
| 56 | Focal Onset | Trunk Twisting. Jerks. Aware | Multifocal Poly-spikes |  | Generalized Jerks |
| 57 | Focal, Bilateral | Vertiginous. TCM | Focal, Bilateral |  | Tiredness. Clonus |
| 58 | Focal, Bilateral | Automatism. TCM |  |  | TCM |
| 59 | Focal, Bilateral | Right Jerks, Jacksonian TCM, Unaware |  |  | Jerks. TCM |
| 60 | Focal, Bilateral | “Aura”. GTC | Focal, Bilateral |  | TCM and Aware |
| 61 | Focal, Bilateral | Dystonic Limb then Unconsciousness |  | Parieto-temporal Infarct | TCM |
| 62 | Focal, Bilateral | Hyperkinetic Movements |  |  |  |
| 63 | Focal, Bilateral | TCM |  |  |  |
| 64 | Focal, Bilateral | Automatism. TCM |  |  |  |
| 65 | Focal, Bilateral | Dystonic Posturing then TCM |  |  |  |
| 66 | Focal, Bilateral | Blank Spells. Tonic Seizure |  |  |  |
| 67 | Focal, Bilateral | Micropsia then LOC |  |  |  |

Abbreviation: TCM: tonic clonic manifestation. LOC: Loss of consciousness. ED: Epileptiform Discharge. UL: Upper Limb
